# Tyrosinase-Based Biosensors for Selective Dopamine Detection

**DOI:** 10.3390/s17061314

**Published:** 2017-06-07

**Authors:** Monica Florescu, Melinda David

**Affiliations:** Faculty of Medicine, Transilvania University of Brasov, 500019 Brasov, Romania; melinda.dav@gmail.com

**Keywords:** cobalt (II)-porphyrin, tyrosinase, electrochemical biosensors, dopamine

## Abstract

A novel tyrosinase-based biosensor was developed for the detection of dopamine (DA). For increased selectivity, gold electrodes were previously modified with cobalt (II)-porphyrin (CoP) film with electrocatalytic activity, to act both as an electrochemical mediator and an enzyme support, upon which the enzyme tyrosinase (Tyr) was cross-linked. Differential pulse voltammetry was used for electrochemical detection and the reduction current of dopamine-quinone was measured as a function of dopamine concentration. Our experiments demonstrated that the presence of CoP improves the selectivity of the electrode towards dopamine in the presence of ascorbic acid (AA), with a linear trend of concentration dependence in the range of 2–30 µM. By optimizing the conditioning parameters, a separation of 130 mV between the peak potentials for ascorbic acid AA and DA was obtained, allowing the selective detection of DA. The biosensor had a sensitivity of 1.22 ± 0.02 µA·cm^−2^·µM^−1^ and a detection limit of 0.43 µM. Biosensor performances were tested in the presence of dopamine medication, with satisfactory results in terms of recovery (96%), and relative standard deviation values below 5%. These results confirmed the applicability of the biosensors in real samples such as human urine and blood serum.

## 1. Introduction

The detection of dopamine (DA) levels in physiological media, is gaining interest as dopamine is an important neurotransmitter which is linked to a large variety of medical conditions [1]. Selective identification of DA concentrations allows the monitoring of Parkinson's and Alzheimer's diseases, attention deficit hyperactivity disorder (ADHD), and schizophrenia [2,3,4,5], and the hormone is becoming accepted as a prognostic biomarker [6]. DA is one of the most important catecholamines, having an important role in the functionality of the central nervous system (CNS), but also influencing the hormonal, renal and cardiovascular systems [7], due to its mobility through the human body [8,9,10]. The treatment of some of the aforementioned diseases is performed with medication that blocks or activates corresponding receptors, and continuous monitoring in patients is required. Herewith, analysis and control of neurotransmitters is of great importance in the field of neuroscience, where current treatment is arbitrary, mainly based on clinical observations of the patient. For quantification, the most widely-used methods for neurotransmitter detection are in vitro methods like high performance liquid chromatography (HPLC) [11], absorption spectroscopy [12], where both methods need special laboratory setups and are time-consuming; or in vivo imaging techniques, which are very expensive [13]. Electrochemical sensors offer a real-time response with high sensitivity and selectivity, due to the electro-active nature of DA [14]. 

One of the problems for the electrochemical detection of dopamine is the co-existence of many interfering compounds in biological systems which can be oxidized at similar potentials, resulting in an overlap of electrochemical responses. The most important interferent of DA is ascorbic acid (AA), a cofactor of multiple enzymes [15]. To overcome these difficulties, different chemically modified electrodes with diamond nanostructures and nanoparticles, functional groups, or polymers, have been used together with various electrochemical techniques, as summarized in the reviews [16,17,18]. Biosensors based on graphene and carbon nanotubes (CNT)’s modified electrodes exhibit good analytical performances [19]. Porphyrins are pyrrole-based polymers, containing redox sites, which can easily be substituted by metallic ions, such as cobalt [20]. They are also known to be important ligands for biochemical interactions, having multiple properties (ranging from optical to thermodynamic and physicochemical), that allow them to be used for electrochemical (bio)sensing [21]. 

Biosensors also offer a real-time response with higher sensitivity and selectivity. According to the literature, tyrosinase biosensors are mainly used for the detection of phenolic compounds by monitoring the reduction signal of the biocatalytically-produced quinone species [22]. This is possible due to the fact that tyrosinase (Tyr) is a copper-containing enzyme, which, in the presence of oxygen, catalyzes two different reactions: (1) the ortho-hydroxylation of monophenols to o-diphenols and (2) the oxidation of o-diphenols to o-quinones [23]. The enzyme immobilization method must be chosen in a way that improves the catalytic properties of the biosensors [24]. Previous reports have described graphene-based tyrosinase biosensors for 2,4-dichlorophenol, and bisphenol A detection [25,26]. For the detection of L-phenylalanine, a boron-doped diamond (BDD) electrode modified with tyrosinase immobilized on polyaniline doped with polyvinyl sulfonate composite films has been used [27]. Boron-doped diamond nanoelectrode arrays (BDD-NEAs) were used for ultrasensitive DA detection by Dincer et al. [28], while glassy carbon electrodes modified with carbon black nanoparticles offered competitively good results [29]. The use of enzyme-based biosensors for the bio-catalytic oxidation of DA, allows for relatively large oxidation potentials to be overcome during direct oxidative detection and electrode surface passivation by the phenoxy radicals (dopachrome); highly selective dopamine measurement can be achieved because tyrosinase is only specific for dopamine, whereas ascorbic acid is not a substrate of tyrosinase. Electrochemically pretreated and activated carbon/tyrosinase/nafion-modified glassy carbon electrodes were developed, as reported in [30]. Bio-composite coatings consisting of poly(3,4-ethylenedioxythiophene (PEDOT) polymer and tyrosinase were electrodeposited onto conventional size gold electrodes and microelectrode arrays using sinusoidal voltages for dopamine and catechol electroanalysis [31]. A composite Pt/PEDOT-PB modified electrode was also successfully tested for DA determination in the presence of ascorbic acid (AA); however, a bio-component was lacking [32]. An electrode system consisting of electrodeposited poly(indole-5-carboxylic acid) film modified with covalently bound tyrosinase, for selective detection of DA in the presence of AA and uric acid (UA) as interfering compounds was also reported [33]. To the best of our knowledge, tyrosinase activity in combination with metalloporphyrin film has not yet been studied.

In this paper, tyrosinase-based biosensor development for sensitive and selective dopamine detection was described. A film of cobalt (II) porphyrin (CoP) with electrocatalytic activity, which acts both as electrochemical mediator and enzyme support, was adsorbed onto a clean gold surface, and further modified with Tyr using a cross-linking agent (CoP-Tyr-biosensor). This combination provided an efficient immobilization matrix for tyrosinase which potentially reduced the distance from the redox-center of tyrosinase to the CoP film and promoted faster electron transfer. The characteristics of the CoP film were morphologically and electrochemically analyzed and CoP-Tyr-biosensor performances were compared to the performances of the CoP-based sensor for the detection of DA, as CoP film also has electrocatalytic activity. The electrochemical characteristics and biosensor performances in terms of selectivity, sensitivity and stability, were discussed. Both CoP-sensors and CoP-Tyr-biosensors were tested in the presence of dopamine medication that is used as treatment for patients with low blood pressure [10].

## 2. Materials and Methods

### 2.1. Materials

All reagents used were of analytical grade. Tyrosinase from mushroom (Tyr) (hydrolyzed powder, ≥1000 U/mg), glutaraldehyde 25% (GA), bovine serum albumin (BSA), dopamine hydrochloride (DA), ascorbic acid (AA), 2,3,7,8,12,13,17,18-octaetil-21*H*,23*H*-cobalt porfirine (II) (CoP), chloroform, and ethanol were purchased from Sigma Aldrich, Germany. A dopamine hydrochloride solution (5 mg/mL) for 10 mL intravenous injections was used as a real sample and was purchased from pharmaceutical company Zentiva. All experiments were performed in neutral sodium phosphate buffer 0.1 M NaPB pH = 8.0. Dajhan LabTech deionized water (resistivity ≥18 MΩ·cm) was used for the preparation of all solutions. Working solutions of DA and AA were freshly prepared before measurements. All experiments were performed at room temperature (22 ± 1 °C) and all electrodes were kept in buffer solution at ~4 °C in a refrigerator, in between measurements. 

### 2.2. Instrumentation

All electrochemical measurements were carried out in a conventional electrochemical cell containing three electrodes. Gold bulk electrodes covered in Teflon (area 0.00785 cm^2^, eDAQ Pty. Ltd., Denistone East, New South Wales, Australia) were used as working electrodes, with a platinum foil as a counter electrode, and a Ag/AgCl electrode (3.5 M KCl) as reference. Chrono-amperometric, electrochemical impedance and voltammetric measurements (differential pulse and cyclic voltammograms) were performed by using a PalmSens3 electrochemical sensor interface (Palm Instruments BV, Houten, The Netherlands) controlled with PSTrace 4.8 software. Differential pulse voltammograms were recorded in different conditions in the presence and absence of the enzyme, where the constant amplitude of the pulse was set to 50 mV (pulse height) for 0.2 s (pulse width), at a scan rate of 10 mV·s^−1^. For impedance measurements, an rms perturbation of 10 mV was applied over the frequency range 50 kHz–0.1 Hz, with 10 frequency values per frequency decade. The obtained spectra were recorded at a potential of −0.2 V vs. Ag/AgCl, and plotted in the form of complex plane diagrams (Nyquist plots) using electrochemical impedance spectroscopy (EIS) Spectrum Analyzer 1.0 software [34]. The surface morphology of the 2,3,7,8,12,13,17,18-Octaethyl-21*H*,23*H*-porphine cobalt(II) thin film was investigated by atomic force microscopy (AFM NT-MDT model NTEGRA PRIMA EC). The images were taken in semi-contact mode with “GOLDEN” silicon cantilever (NCSG10, force constant 0.15 N/m, tip radius 10 nm).

### 2.3. CoP-Film Preparation and Construction of Tyrosinase Biosensor

A clean gold electrode was modified with CoP film by immersion in 0.5% solution of CoP dissolved in chloroform, for 10 min. For the next 10 min, the adsorbed film was dried in air and deposited in buffer solution in order to avoid the interaction of the porphyrin film with gas molecules [35]. For the Tyr-based biosensor, after drying the CoP film, an aqueous solution containing 1% Tyr and 4% BSA, was mixed with cross-linking agent 2.5% GA and pipetted onto the electrode surface; thus a concentration of 0.67% Tyr was immobilized. The enzyme layer was left to dry for 10 min, washed, and stored in buffer solution in between measurements.

## 3. Results and Discussions

### 3.1. Characterization of the CoP-Tyr Biosensor

The monitoring of neurotransmitter levels is vague, highlighting the need for rapid and selective tools. Electrochemical sensors have some limitations, especially due to the lack of resolution between DA and other electroactive species coexisting in the cerebral system (the concentrations of some interferent species, such as AA, are much higher than that of DA in the CNS). Biosensor performances offer here an advantage towards selectivity, sensitivity, and surface morphology. This, together with electrochemical characterization, was performed as follows.

#### 3.1.1. Surface Morphology Analysis of CoP Film

**Figure 1:**
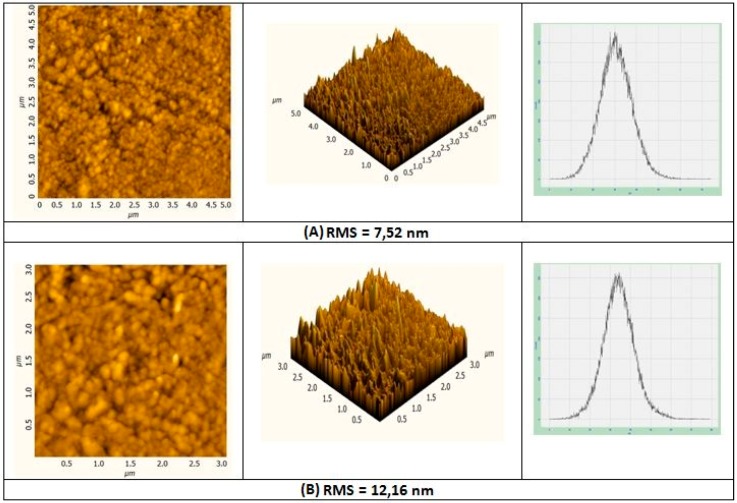
Atomic force microscopy (AFM) image monitoring of 0.5% CoP film growth upon adsorption from (**A**) 7.52 nm to (**B**) 12.16 nm Topography-phase contrast is shown in 2D, topography in 3D, and a corresponding histogram depicting height distribution.

Atomic Force Microscopy (AFM) was employed to analyze the surface morphology of the CoP-film. Surface topography is usually described by amplitude parameters such as average roughness and root mean square roughness (RMS) [36]. It was in our interest for the thin film to offer a good support for enzyme immobilization. In this context, it was important for the film to have a large number of active adsorption sites. This can be appreciated from the film roughness. The degree of particle deposition upon a surface, is given by an increasing value of the RMS, as shown in Figure 1. For a RMS of 12.16 nm, the peaks and valleys are more pronounced, offering accessible binding sites on the films for enzyme layer. However, the CoP film was evenly distributed, as seen from the histograms.

#### 3.1.2. Electrochemical Characterization of CoP Film

**Figure 2:**
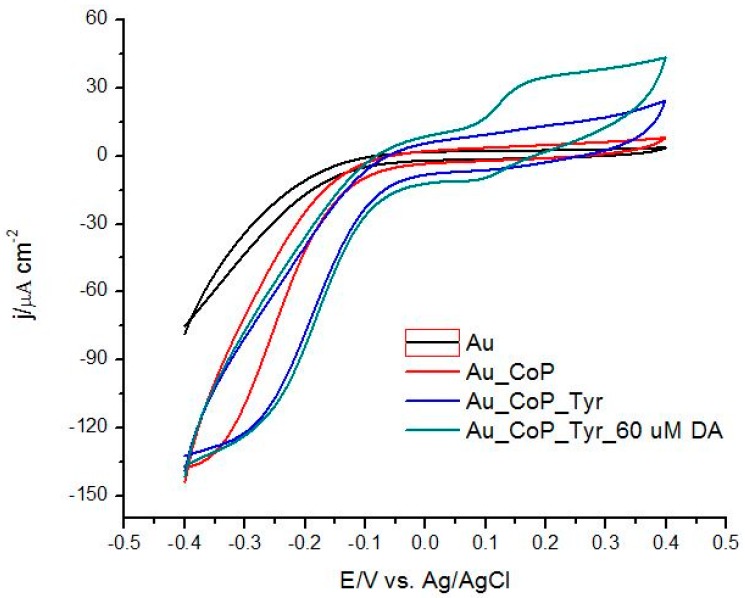
Cyclic voltammetry for the Au, Au-CoP, Au-CoP-Tyr modified electrode in 0.1 M NAPB, pH 8.0, v = 50 mV·s^−1^, with an addition of 60 µM dopamine (DA).

Cyclic voltammetry (CV) offers an insight into the overall characteristics of a sensor material. Figure 2 illustrates the voltammograms of the modified Au electrode in NaPB, pH 8.0, at a scan rate of 50 mV·s^−1^. It can be said that responses of the modified electrodes CoP and CoP-Tyr are bigger compared to bare gold electrode, which may be attributed to the increase in surface area of the modified electrodes. Voltammograms obtained with the CoP-Tyr-biosensor in presence of two different DA concentrations showed a considerable increase in current intensity for the peaks of the DA redox reaction. This suggested an increase in the electron transfer rate. Figure 2 reveals a very well defined anodic peak assigned to the oxidation of DA, which was paired with the corresponding smaller reduction peak. This suggested that a quasi-reversible redox reaction of DA occurred at the CoP-sensor, which was associated with its oxidation product associating/dissociating from the CoP film.

**Figure 3:**
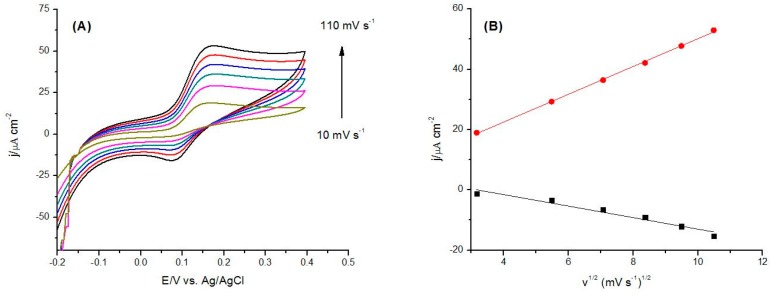
(**A**) Cyclic voltammograms in 0.1 M NaPB. pH 8.0, containing 100 µM DA at different scan rates: 10, 30, 50, 70, 70 and 110 mV·s^−1^; (**B**) the plots of both peak currents I_pa_ and I_pc_ vs. the square root of the scan rate.

In order to characterize the mechanism of the electrode reaction at the CoP film, the relationship between scan rate and peak current during CVs was studied, as shown in Figure 3A. There was a considerable increase in the current intensity for the oxidation peak of DA, with an increasing scan rate. This suggested an increase in the electron transfer rate. However, the oxidation peak potential (0.15 V) was almost unchanged, exhibiting a linear relationship with the square root of the scan rates over the range 10–110 mV s^−1^. The same linear range was observed for the reduction peak at 0.08 V. The linear relationship of the square root of the scan rate with the values of the anodic peak current ($I_{pa}$) and cathodic peak current ($I_{pc}$) can be seen in Figure 3B. Taking into account the linear regression equations below, the influence of scan rate explained the electrode process in terms of a diffusion controlled reaction (mass transport).

(1) $$I_{pa}\;\left(\mu A\right)=0.036\;v^{\frac{1}{2}}+0.032\;{\left({\mathrm{mV}\;s}^{-1}\right)}^{1/2};\;R^{2}=0.998$$

(2) $$I_{pc}\;\left(\mu A\right)=-0.014\;v^{\frac{1}{2}}-0.047\;{\left({\mathrm{mV}\;s}^{-1}\right)}^{1/2};\;R^{2}=0.946$$

#### 3.1.3. Electrochemical Impedance Spectroscopy 

**Figure 4:**
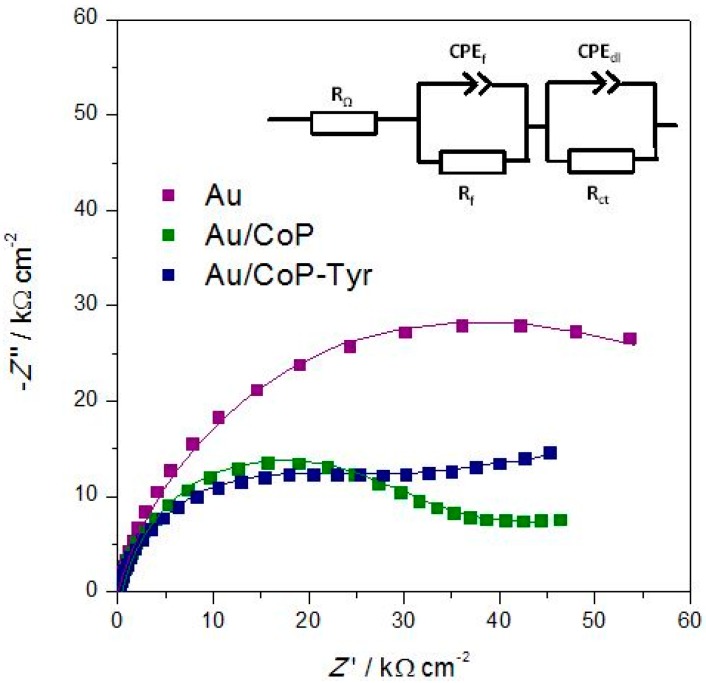
Complex plane plots for Au, Au/CoP and Au/CoP-Tyr in 0.1 M NaPB pH 8.0, at −0.2 V vs. Ag/AgCl with the inset showing the equivalent electrical circuit used to fit the spectra.

EIS was used to characterize the bulk and electrode/electrolyte interface phenomena, providing information about electron transfer and charge polarization. It was employed to identify interfacial changes after CoP film deposition and Tyr entrapment, similar to a layer-by-layer (LbL) structure formation on the electrode surface. Figure 4 shows the complex plane representation of the fitted impedance spectra acquired for Au, Au/CoP and Au/CoP-Tyr at a potential applied during measurements of −0.2 V vs. Ag/AgCl. The potential value was chosen to highlight the modifications of the electrode-electrolyte interface phenomena with each layer deposition, taking into consideration that the electrode material was Au.

The spectra were fitted using an equivalent electrical circuit shown in the inset of Figure 4, and consisted of a cell resistance, representing the electrical resistance of the cell and electrolyte solution (R_Ω_), in series with two parallel combinations. The first parallel combination was associated, in all situations, with a CoP or CoP-Tyr film modified with an Au electrode/electrolyte solution interface and consisted of a charge transfer resistance (R_ct_) and a double layer non-ideal capacitance (CPE_dl_). The Au electrode/film interface (CoP or CoP-Tyr films) introduced in series a second parallel combination of the film charge-transfer resistance (R_f_) and a non-ideal capacitance (CPE_f_). Both non-ideal capacitances were represented by constant phase elements (CPE), according to the equation:

(3) $$CPE=\;{\left[{\left(Ci\omega \right)}^{\alpha }\right]}^{-1}$$

where C is the ideal capacitance, ω the radial frequency and the exponent α, which reflects the surface uniformity.

Table 1 shows the values of the circuit components obtained by fitting the experimental spectra to the electrical equivalent circuit. The value of the electrical resistance of the electrolyte solution and electrical contacts remained almost constant throughout each deposition step, R_Ω_ ≅ 18 Ω·cm^2^. The CoP film and the enzyme layer contribution were shown by the high CPE_f_ values, ranging between 956.3 and 901.6 µF·cm^−2^·s^α−1^. This indicated a charge accumulation at the electrode/film interface. The film resistance value increased from 0.78 kΩ·cm^2^ for CoP (conductive), up to 1.42 kΩ·cm^2^ after enzyme immobilization (less conductive layer), which was in concordance with CPE_f_ values. The high average value of α_f_ ≅ 0.97 reflected the uniformity and smoothness of both enzyme and CoP films, in accordance with the AFM images. At the electrolyte interface, R_ct_ values were higher for the bare electrode (4.64 kΩ·cm^2^) and started decreasing with each deposited layer: from 4.64 kΩ cm^2^ for bare Au, to 2.09 kΩ·cm^2^ for Au-CoP and 2 kΩ·cm^2^ after enzyme entrapment, which was attributed to a higher electron transfer through interface. The value of CPE_dl_ increased from 29.4 µF·cm^−2^·s^α−1^ for bare Au, to 30 µF·cm^−2^·s^α−1^ for CoP film, and doubled for the enzyme layer. This suggested that the adsorption of both materials led to changes in space charge polarization. The values of α_dl_ ranging from 0.80 to 0.87 suggested that the interface changed after each adsorption step.

**Figure 5:**
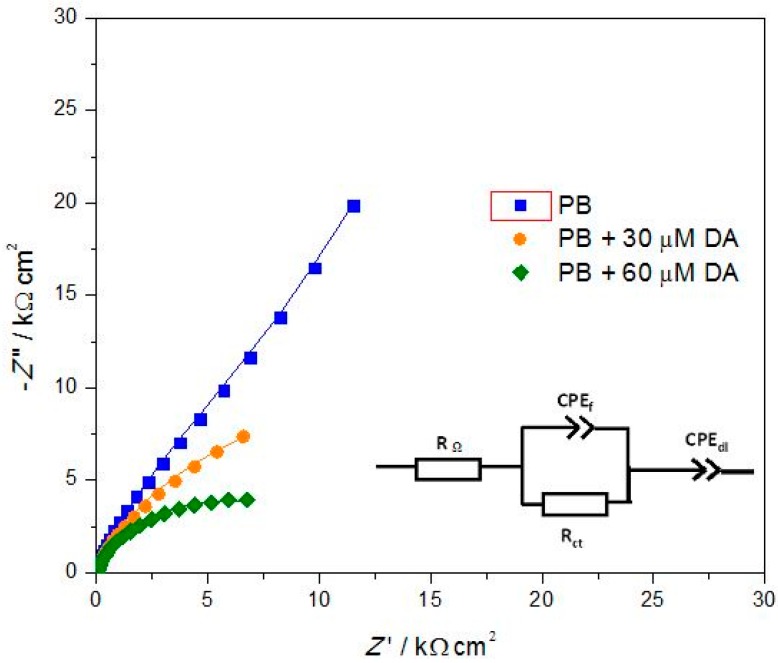
Complex plane plots for Au/CoP-Tyr in 0.1 M NaPB pH 8.0, with the successive addition of 30 and 60 µM DA at 0.1 V vs. Ag/AgCl, with the inset showing the equivalent circuit used to fit the spectra.

EIS was also used to characterize the electrode/electrolyte interface phenomena, in the presence of dopamine. Figure 5 shows the complex plane representation, with a 0.1 V potential applied during measurements, of the impedance spectra acquired for Au/CoP-Tyr in 0.1 M NAPB, pH 8.0 containing 30 and 60 µM DA. The semicircle diameter of this Nyquist plot reflected the electron transfer resistance (R_ct_), which refers to current flow produced by the reactions at the interface, and was found to be lower when the DA concentration increased, suggesting the bio-catalytic activity of tyrosinase at the surface of the biosensor towards the oxidation of DA. The spectra were fitted using an equivalent electrical circuit shown in the inset of Figure 5. This consisted of a cell resistance, R_Ω_, in series with a parallel combination of a charge transfer resistance, R_ct_, through the Au-CoP/Tyr film interface, and a double layer capacitance (CPE_f_), represented as a constant phase element, which resulted from charge being stored in the double layer at the interface, in high and intermediate frequency regions. A further CPE element (CPE_dl_), in series with the parallel combination, was used to monitor the capacitive behavior of the upper immobilized enzyme layer (Tyr cross-linked with glutaraldehyde), in contact with the electrolyte in the low frequency region, which significantly varied in the presence of DA.

Table 2 shows the values of the circuit components obtained by fitting the experimental spectra to the equivalent electrical circuit for DA oxidation. The cell resistance kept an almost constant value around 5 Ω·cm^2^. As expected, the value of the charge transfer resistance R_ct_ keeps decreasing for each DA addition, from 58.57 kΩ·cm^2^ in the absence of DA, to 6.99 kΩ·cm^2^ in the presence of DA, indicating the conducting properties of the Au and CoP film. The capacitance of the modified electrodes with electrocatalytically CoP film depends mainly on the surface area accessible to the electrolyte ions and redox species, which depends in turn on the specific surface area, pore-size distribution and shape. The increase in both the double layer capacitance CPE_f_ (61.07 to 100.01 μF·cm^−2^·s^α−1^) and the $\alpha_{dl}$ values ranging from 0.71 to 0.82 indicated a charge accumulation at the Au-CoP/Tyr layer interface, influenced by the oxidation of DA. At the upper Tyr layer /electrolyte interface, capacitance values CPE_dl_ also increased with DA concentration, doubling in value from 300.63 (in the absence of DA) to 620.50, and 665.47 μF·cm^−2^·s^α−1^ respectively, for 30 and 60 µM DA. With DA and dopa-quinone molecules accumulating at the electrode/film interface, α_f_ values decreased.

#### 3.1.4. The Role of CoP in Dopamine Oxidation

The rate of the electrochemical reactions was significantly influenced by the nature of the electrode surface. Porphyrins are less widely used as a surface modifier or electrochemical mediator in (bio)sensors and their interaction with different analytes are less well-studied. Metalloporphyrins (porphyrin systems with metallic ions) have low energy excitations in the visible spectral region, and they also accept or donate electrons easily [37]. The two-dimensional geometry of porphyrins and their electronic structure both promote very rapid and vectorial electron transfer, and thorough interaction of these macrorings with analytes [38]. It has been highlighted that two fundamental cooperative effects can take place in the sensing phenomenon, and are the main determinants of the performances of chemical sensors based on porphyrins: weak interactions (such as Van der Waals or London forces and hydrogen bonding) and the coordination of analytes [39].

The central metal of the metalloporphyrin affects sensing, as dopamine oxidation to dopaquinone can be performed using both transition metals, as well as catalysis by enzymes (e.g., tyrosinase). For CoP-sensors, the aromatic-stacking and electrostatic attraction between positively-charged dopamine (protonated amine group at physiological pH) and negatively-charged porphyrin can accelerate the electron transfer, while weakening AA oxidation (the main interferent on physiological samples) on the porphyrin-functionalized gold-modified electrode. For CoP-Tyr-biosensor, the CoP film acts as a mediator to enhance the direct electron transfer between the enzyme and Au electrodes, which is usually prohibited due to the shielding of redox active sites by the protein shells. Mediators are widely used to access the redox center of the enzyme, and thus act as electron shuttles.

The following characteristics recommend the use of metalloporphyrins:

1. Electrocatalytic activity toward dopamine oxidation (enhancing the electronic conductivity and promoting electron transfer rate between the DA and electrode surface) in electrochemical sensor development, and
2. Electrochemical mediator activity during enzyme-catalyzed oxidation of dopamine (enhancing electronic conductivity and acting as charge carriers) and
3. Support for enzyme immobilization for biosensor development.

**Scheme 1:**
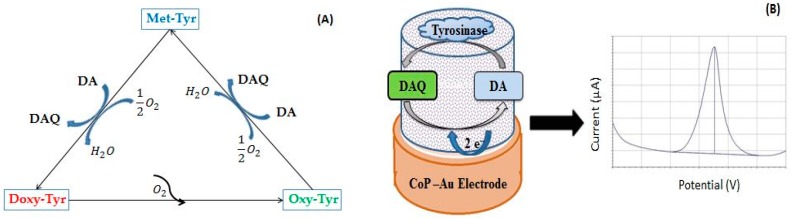
(**A**) Tyrosinase forms during enzyme catalysis of dopamine at CoP-Tyr-biosensor and (**B**) detection principle at the CoP-Tyr biosensor surface.

With CoP-sensors, dopamine can be easily electrocatalytically oxidized at the CoP film to form dopamine quinone (DAQ) which can be reduced at the electrode surface when a potential is applied to the electrode, after the exchange of two electrons (and two protons) to produce a Faradaic current [40]. In the case of biosensors, during the DA oxidation steps, the oxidation states of the copper atoms of tyrosinase change to give different forms of the enzyme [23]. Native tyrosinase occurs mainly as met-tyrosinase (Met-Tyr) in which a hydroxyl ion is bound to the two copper ions and both copper ions are in the Cu(II) oxidation state; this form, in the presence of oxygen, catalyzes the oxidation of catechols like DA to DAQ with H_2_O production. During this process, Met-Tyr is reduced to deoxy-tyrosinase (Doxy-Tyr) in which both copper ions are in the Cu(I) oxidation state. Doxy-Tyr binds oxygen to generate Oxy-Tyr, which is reduced to Met-Tyr while it catalyzes the oxidation of DA to DAQ (Scheme 1A). DAQ is further reduced at the electrode surface (Scheme 1B).

### 3.2. Optimization of Experimental Conditions for DA Detection

#### 3.2.1. Influence of pH

In order to enhance the selective DA detection in the presence of AA at the CoP-film surface, the influence of the pH of the supporting electrolyte on the electrochemical activity of the CoP-sensors was studied. The current response obtained from the enzyme-catalyzed reaction presented a maximum value and maximum peaks separation at pH 8.0, which was chosen for all measurements.

#### 3.2.2. Influence of CoP Concentration

The effect of cobalt (II)-porphyrin compound concentrations in the solution used for CoP film deposition on an Au surface was studied, and a concentration of 0.5% in chloroform provided better results, thus this concentration value was selected.

#### 3.2.3. Parameters of the Detection Method

**Figure 6:**
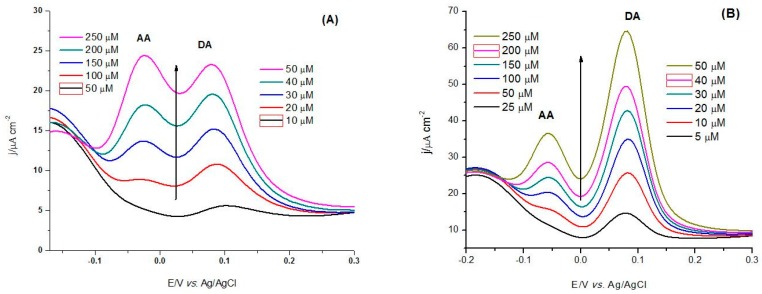
Differential pulse voltammograms with 50 mV pulse height and 0.2 s width, for the simultaneous variation of 5–50 µM DA and 25–250 µM AA in 0.1 M NAPB, pH 8.0, for: (**A**) the CoP-sensor and (**B**) the CoP-Tyr-biosensor.

Since differential pulse voltammetry (DPV) is more sensitive for cyclic voltammetry, it was used to detect DA (in the absence and presence of AA). In order to increase the sensor response, pretreatment steps were employed, immediately followed by the measurement step. All steps were performed in the electrolyte solution containing the analytes (DA and AA). The effect of modifying the electrode surface with CoP film was noticed in DPV since the separated DA and AA peaks appeared at lower potentials: at 0.08 V for DA and at −0.03 V for AA (compared with 0.15 V for both AA and DA with bare electrodes, results not shown). Optimized parameters were determined for both selective and sensitive DA detection (Figure 6A), as well as to avoid electrode surface passivation by the phenoxy radicals (dopachrome). Thus, the optimized pretreatment settings of the CoP-sensors towards DA detection were: a conditioning potential of −1.2 V for a period of 20 s, followed by 40 s of a preconcentration step at −0.5 V. The pretreatment influence on the CoP-Tyr-biosensor response was studied, to better discriminate between the AA and DA peaks, since the peaks of AA and DA were slightly overlapping without any pretreatments. In the presence of the enzyme, the conditioning potential was no longer necessary, since it did not influence the peak potentials (data not shown). The variation of the preconcentration potential was studied, and a value of −1.3 V for a fixed time of 40 s was chosen, since it clearly assisted in discriminating the AA and DA peaks (Figure 6B). Since AA (which is not a substrate of tyrosinase) can diffuse to the electrode surface, we can concluded that the use of negative potentials in the pretreatment steps, was necessary to better discriminate between the AA and DA peaks for both the CoP-sensor and the CoP-Tyr-biosensor. Positive potentials were also tested, but no discrimination between the two peaks was observed.

### 3.3. Electrocatalytic Oxidation of Dopamine

#### 3.3.1. Analytical Parameters of the CoP-Tyr-Biosensor vs. the CoP-Sensor

**Figure 7:**
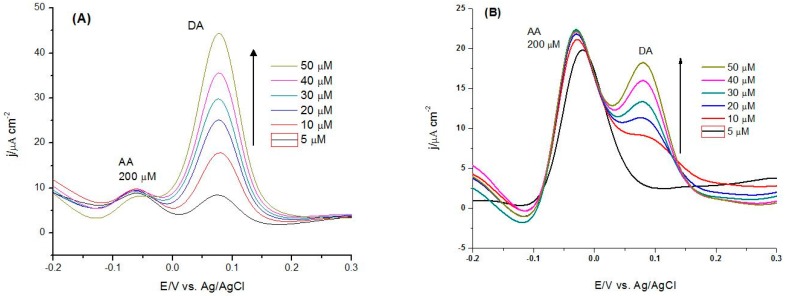
Differential pulse voltammograms with 50 mV pulse height and 0.2 s width, in 0.1 M NAPB, pH 8.0, v = 50 mV s^−1^ of: (**A**) the Au-CoP-Tyr biosensor, and (**B**) the Au-CoP- sensor for 200 µM AA and 5–50 µM DA.

DPV of DA at the CoP-sensor showed that the DA peak increased with increasing concentration. The determination of DA performed under optimized conditions exhibited a linear response from 10 to 50 µM, a sensitivity of 0.577 ± 0,041 µA·cm^–2^·µM^–1^, and a detection limit of 0.98 µM [limit of detection (LOD) = 3 × SD/S, where SD was the standard deviation of the blank and Swas the sensor sensibility). All of the calibration curves were obtained in triplicate. Simultaneous increasing concentrations for AA and DA (5–50 µM DA and 25–250 µM AA) were also monitored, and the data is presented in the Figure 6A. In this case, the CoP-sensor performances towards DA were slightly decreased to a sensitivity of 0.443 ± 0.01 µA·cm^−2^·µM^−1^, and an increased detection limit of 3.13 µM (R^2^ = 0.996). The CoP modified sensor reached a sensitivity of 0.561 ± 0.01 µA·cm^−2^·µM^−1^ and a detection limit of 2.46 µM (R^2^ = 0.997) for DA, when 200 µM AA concentration was kept constant (Figure 7B). Since in physiological conditions (the central nervous system), the concentrations of AA can be 100–1000 times higher than those of DA [41], all experimental data was carried out with a higher ratio of AA to DA.

The sensor performances were also tested in the presence of dopamine hydrochloride (5 mg/mL) medication, which is used in intravenous infusions. The data is presented in comparison to the CoP-Tyr-biosensor in Chapter 3.4.

DPV was also used as a sensitive and selective detection method for DA in the presence of AA using the CoP-Tyr-biosensor. The peak potential for AA was in the negative region at −0.07 V while that of DA was at 0.06 V, maintaining a difference of 130 mV between the two peaks. Table 3 summarizes the analytical performances of both CoP and CoP-Tyr (bio)sensors.

**Figure 8:**
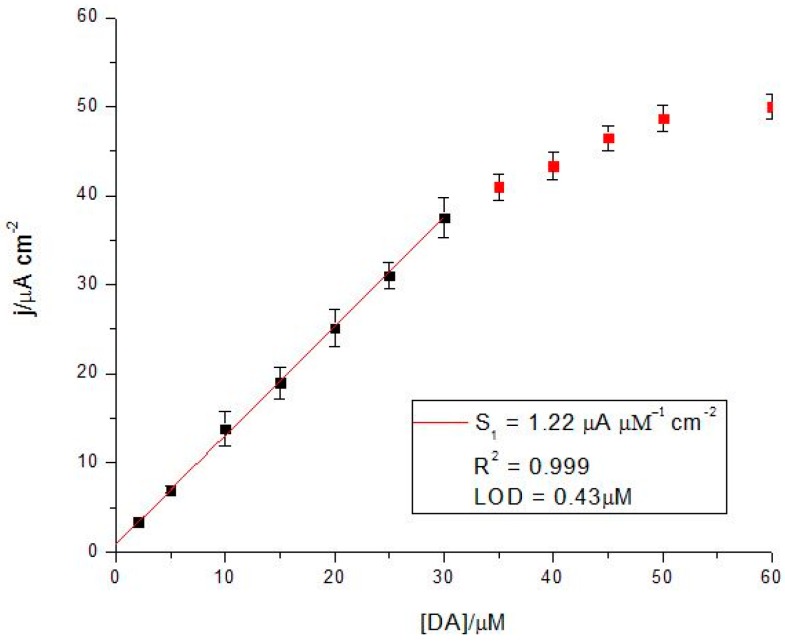
Calibration plot of Au-CoP-Tyr biosensor for 2–60 µM DA in 0.1 M NAPB, pH 8.0, v = 50 mVs^−1^.

The biosensor performance was tested under the established conditions. Figure 8 shows a linear response of the CoP-Tyr-biosensor for DA for the range 2 to 30 µM, with a sensitivity of 1.22 ± 0.02 µA cm^−2^ µM^−1^ and a detection limit of 0.43 µM. The average sensitivity of the biosensor was considerably higher in comparison to the CoP-sensor (R^2^ = 0.997). All the calibration curves were obtained in triplicate. Thus, for the CoP-Tyr-biosensor, the peaks of DA were much higher than that at the CoP-sensor. Figure 6B presents the CoP-Tyr-biosensor response for simultaneous increasing concentrations of AA and DA. The concentration of DA was varied as previously, while the concentration of AA was varied from 25 to 250 µM (50 µM per injection). It can be seen that the peak currents for the two analytes increased linearly with their concentrations. The biosensor performances for DA remain at a sensitivity of 1.21 ± 0.03 µA cm^−2^ µM^−1^ with a detection limit of 0.55 µM. The change of AA concentration did not have a significant influence on the peak current and the peak potential of the DA. Figure 7A shows the response for DA in the presence of a fixed concentration of 200 µM AA, with a slightly lower sensitivity of 1.21 ± 0.02 µA cm^−2^ µM^−1^ with a detection limit of 0.52 µM. The changes of AA peaks were at the relative standard deviation (RSD) level of individual voltammetric measurements (here about 2.1%). The peaks were well separated on both situations when the CoP-Tyr-biosensor was used; around 130 mV, which is enough for avoiding undesired interferences. By comparison with the CoP-sensor (Figure 7B), the separation between peaks is bigger and DA peaks are much higher. These results were reflected in the higher sensitivity and lower LOD for CoP-Tyr-biosensor towards DA in the presence of AA.

The values of the apparent Michaelis–Menten constant K_M_ and the maximum current density J_max_, (corresponding to tyrosinase saturated with dopamine) were estimated by a Lineweaver–Burk type plot. Values of 155.52 µA cm^−2^ for the maximum current and 92.68 µM for K_M_ were obtained, which are in accordance with values obtained in the literature for immobilized tyrosinase (60–200 µM). Generally, the Michaelis–Menten approach applied to the immobilized enzymes typically yields increased values of K_M_. The effect of surface confinement on the kinetics of bound enzymes is mainly related to the diffusion limitation of the substrate to the reaction centers, by virtue of the immobilization itself (spatial limitation). Smaller K_M_ values suggest a higher affinity or binding strength between the immobilized enzyme and its substrate dopamine, that can overcome the negative effect of immobilization and possible conformational changes of the enzyme.

#### 3.3.2. Biosensor Stability

**Figure 9:**
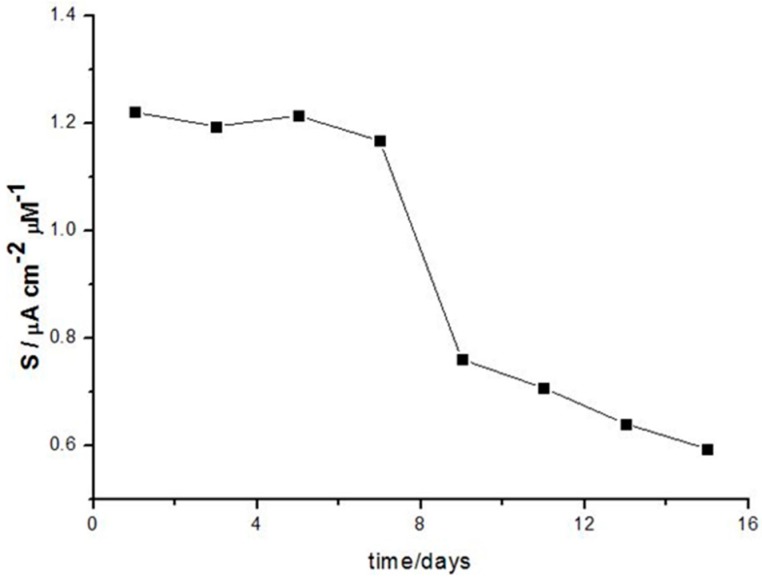
Biosensor long term stability for DA detection.

The long term stability of our biosensors was evaluated by repeated measurements on different days over a timeframe of two weeks, determining its sensitivity for DA detection, as shown in Figure 9. The biosensor kept its activity up to 95% in the first week, dropping to less than 50% on the 9th day, and decreasing subsequently. In comparison to the CoP sensor, whose sensitivity dropped after two days (data not shown), the CoP-Tyr biosensor had better stability, with the sensitivity keeping initial values for almost a week. In between measurements, the biosensor was kept in buffer solution in the refrigerator for ~4 °C.

#### 3.3.3. Real Sample Detection

Sensor and biosensor performances were tested in the presence of dopamine hydrochloride medication (5 mg/mL) with an equivalent of 26.36 mM DA, which is usually used in blood pressure treatments. Aqueous dilutions up to 1000–10,000 times (depending on the working electrode type CoP, respectively CoP-Tyr) were prepared. The concentration of dopamine from the vial (C_x_) was determined using standard addition method (SAM) and its recovery was calculated. After adding the sample, three known concentrations of DA were added, in order to extrapolate the value for C_x_. All of the measurements were done in triplicate. The results are presented in Table 4. Since the relative standard deviation (RSD) values obtained for the biosensor had a value up to 4.7%, this indicated a potential use for the biosensor in real samples, such as blood or urine. The average recoveries were 96% for the CoP-Tyr biosensor and 99.6% for the CoP sensor. The lower value for the CoP-Tyr biosensor recovery can be explained by conformational changes induced by the cross-linking immobilization method, affecting the active site of the enzyme. Also, dopamine and dopamine-quinone diffusion towards the electrode surface can be hindered. Milder enzyme immobilization methods should be considered for future approaches.

Calibration curves of both the CoP-sensor and the CoP-Tyr-biosensor have been also used to determine the sample concentration. The recovery for each found value is presented in Table 5.

The performances of our sensors were also compared with other sensors reported in the literature for dopamine detection, and the results are shown in Table 6.

## 4. Conclusions

The novel Tyr-based biosensor cross-linked on cobalt (II)-porphyrin (CoP) film was fabricated for the sensitive and selective detection of dopamine. AFM images confirmed the presence of a thin film of CoP on the Au sensor surface, offering accessible binding sites for the direct immobilization of the enzyme layer. CoP has been proven to act as an electrochemical mediator during enzyme-catalyzed reaction, CV and EIS measurements showing improved electron transfer. CoP-Tyr biosensor presents high sensitivity and good stability up to seven days. A sensitivity of 1.22 ± 0.02 µA cm^−2^ µM^−1^ and a detection limit of 0.43 µM, with a linear range up to 30 µM were found, comparable with results found in the literature for nanoparticle-based sensors. In the presence of AA as an interferent, there was slight decrease in sensitivity. However, there was a separation of 130 mV between the AA-DA peaks in the DPV plot, which was more than enough to clearly discriminate between the two substances. By comparison with the CoP-sensor, the separation between peaks for the CoP-Tyr-biosensor was bigger, and the DA peaks were much higher. These facts are reflected in the higher sensitivity and lower LOD for the CoP-Tyr-biosensor towards DA in the presence of AA. Both sensors were analyzed in the presence of commercially available dopamine medication, showing good recovery and RSD, but in different linear ranges. These results suggested potential applicability of the biosensors for real samples such as human urine and blood serum.

## Figures and Tables

**Table 1:** Equivalent circuit element values obtained by fitting the impedance spectra from Figure 5 to the corresponding circuit.

Electrode	*R*_f_/kΩ·cm^2^	CPE_f_/μF·cm^−2^·s*^α^*^−1^	*α*_f_	*R*_ct_/kΩ·cm^2^	CPE_dl_/μF·cm^−2^·s*^α^*^−1^	*α*_dl_
Au	-	-	-	4.64	29.40	0.82
Au/CoP	0.785	956.3	0.98	2.09	30.07	0.87
Au/CoP-Tyr	1.420	901.6	0.96	2.00	60.43	0.80

**Table 2:** Equivalent circuit element values obtained by fitting the impedance spectra to the corresponding circuit for DA oxidation.

Electrode	*R*_ct_/kΩ·cm^2^	CPE_f_/μF·cm^−2^·s*^α^*^−1^	*α*_dl_	CPE_dl_/μF·cm^−2^·s*^α^*^−1^	*α*_f_
Au/CoP-Tyr	58.57	61.07	0.71	300.63	0.98
Au/CoP-Tyr 30 µM DA	26.12	90.89	0.74	620.50	0.65
Au/CoP-Tyr 60 µM DA	6.99	100.01	0.82	665.47	0.62

**Table 3:** Analytical performances of both CoP and CoP-Tyr (bio)sensors for DA in the presence (fixed and varying values) and absence of AA.

Analyte	(Bio)sensor	Linearity/µM	S/µA·cm^−2^·mM^−1^	LoD/µM
DA	CoP	10–50	0.57	0.98
	CoP-Tyr	2–30	1.22	0.43
DA and fixed AA	CoP	20–50	0.56	2.46
	CoP-Tyr	10–30	1.21	0.52
DA and AA	CoP	20–50	0.43	3.13
	CoP-Tyr	10–30	1.21	0.55

**Table 4:** Determination of DA in dopamine hydrochloride (*n* = 3) by SAM.

Sensor	DA Specified/µM	DA Added/µM	DA Found/µM	RSD (%)	Recovery (%)
CoP	26.36	0	27.38 ± 0.83	5.9	103.0
	26.36	10	36.57 ± 1.02	5.2	100.5
	26.36	20	46.12 ± 0.53	2.1	99.0
	26.36	30	54.42 ± 0.97	3.2	96.0
CoP-Tyr	3.00	0	3.17 ± 0.32	2.6	105.0
	3.00	2	5.07 ± 0.28	4.0	101.0
	3.00	4	6.24 ± 0.25	2.7	89.0
	3.00	6	8.07 ± 0.59	4.7	89.0

**Table 5:** Determination of DA in dopamine hydrochloride vial (*n* = 3) using the calibration curve from Figure 8.

Sensor	DA Specified/µM	DA Found/µM	$\bar{\mathrm{DA}\;\mathrm{Found}}$/µM	Recovery (%)
CoP	26.36	29.05	27.50 ± 1.45	110
	26.36	26.18		99
	26.36	27.28		103
CoP-Tyr	3.00	3.43	3.10 ± 0.32	114
	3.00	2.79		93
	3.00	3.09		103

**Table 6:** Comparison of analytical parameters of sensors reported in the literature for dopamine detection.

Sensor	Limit of Detection (LoD)/μM	Linear Range/µM	Interference	Recovery (%)	RSD (%)	Ref.
GCE-TCPP/CCG	0.010	0.1–1	AA, UA	~99.0	3.00	[42]
GCE-PPyox/graphene	0.100	25–1000	AA	-	4.30	[6]
GCE-Tyr-Fe_3_O_4_-CHIT	0.006	0.02–0.75	AA	~105.0	5.18	[43]
GCE-GNS-Bi	0.350	1–30	-	98.4	2.20	[44]
GCE-RGO-Pd-NPs	0.230	1–150	AA, UA	98.8	-	[45]
Au-CoP	0.750	10–50	AA	~99.6	4.10	This work
Au-CoP-Tyr	0.430	2–30	AA	~96.0	3.50	This work

TCPP—meso-tetra (4-carboxyphenyl)porphine; CCG—chemically reduced graphene; GCE—glassy carbon electrode; PPyox—overoxidized polypyrrole; Tyr—tyrosinase; CHIT—chitosan; GNS—graphene nanosheets; Bi—bismuth nanoparticles; RGO—reduced graphene oxide; Pd-NPs—palladium nanoparticles.

## Supplementary Materials

Supplementary File 1
