# Supplementary material for: Tyrosinase-Based Biosensors for Selective Dopamine Detection
Source: Sensors (Basel). 2017 Jun 7;17(6):1314. doi: 10.3390/s17061314 (PMC5492229; doi:10.3390/s17061314)
Supplement: Supplementary File 1 [file sensors-17-01314-s001.docx]

**Cover letter**

**Title: Tyrosinase-based biosensor for selective dopamine detection**

**Authors: Monica Florescu, Melinda David**

**Dear Editor,**

Thank you very much for your suggestions. We have modified and add material in the manuscript according with reviewers’ recommendations as follow:

**Reviewer 1**

1. Tyrosinase in the abstract should be written in lower case

-> Done.

2. In the abstract please remove compound in line 11.

-> Done

3. Differential pulse voltammetry (line 14) could be written without capital letters.

-> Done

4. A reduction of the introduction (especially the first paragraph) it is recommended.

-> Done

5. The 3.1 paragraph should be removed since it is not about the sensor characterization

-> It is introducing the following subparagraphs.

6. The EIS part should be better explained

-> Done.

7. The scheme 1 and the paragraph above are not correct: from the scheme 1 it seems

that the turnover of the enzyme is assured by the DA/DAQ molecules; or this is not

true, the O2 is the co-factor. Please carefully revise this scheme.

-> Revised and corrected (Scheme 1A).

8. The paragraph “3.2.3. Parameters of the detection method” should be better explained. Why is there a negative conditioning and preconcentration potential? On which solution is the conditioning potential applied? This high negative potential would not reduce the Co^2+^ cations, and hence reduce their catalytic activity?

-> the use of negative potentials, in the pretreatment steps, was experimentally observed as necessary to better discriminate between the AA and DA peaks for both CoP-sensor and CoP-Tyr-biosensor. Positive potentials were also tested but no discrimination between the two peaks was observed.

In our opinion, to explain using negative potentials in the pretreatment steps we have to take into consideration the combination of several factors which can influence both the working parameters of the sensors and electrocatalytic activity of CoP film. These factors can be: the Co ion is bound inside of the porphyrin molecule forming a complex negatively-charged structure, the pH of the electrolyte must be 8, this influencing both CoP and DA charges, etc..

9. A general scheme of the detection principle should be add in order to facilitate the

understanding of the detection principle.

-> Added (Scheme 1B)

10. The pulse height and width of the DPV should be mentioned in figure captions as

well as in instrumentation part (sub chapter 2.2.) from experimental section.

-> Done

11. For the EIS studies, what redox probe was used? If there is not a mistake, and the

EIS spectra were drawn without a redox probe, how this could be explained?

-> All EIS experiments were done without any redox probe since the probe can be retained in subsequent layer deposition steps. EIS provides a convenient way to examine the interface region without addition of redox probe [CMA. Brett, ECS Transactions, 13(12), 67-80, 2008]. CoP and tyrosinase, as well as DA, have redox activity and promote charge transfer. For each EIS measurements type the applied potential has different value, function on studied phenomena.

12. The 3.3.1 paragraph would benefit from a table with all the information from this

paragraph (LOD, sensitivity etc).

-> Inserted Table 3

-> Renumbered the Tables

13. Regarding the biosensor stability how the sensors were kept for 9 days?

-> Added

14. Please check again the language, some mistakes were found (absorbed instead of

adsorbed in line 83 page 2 etc).

-> Done

**Reviewer 2**

1) Page 1 Line 21-22: Please use instead of the verb “determine” prove or show.

-> Done

2) For the sentence in Page 2 Line 44-45, please provide a reference and remove its repetition

in Page 3 Line 133-134.

-> Done

3) In the introduction, different electrode materials such as carbon-based materials and their

dopamine sensing performances have to be discussed. Please also add the following

references:

a. Lupu *et al.*, Electrochimica Acta, 2009, 54(7), 1932–1938.

b. Dincer *et al.*, Electrochimica Acta, 2016, 185, 101–106.

c. Stoytcheva *et al.*, Current Analytical Chemistry, 2017, 13(2), 89–103.

d. Yang *et al.*, Carbon, 2016, 99, 90–110.

e. Jiang *et al.*, ACS Applied Materials and Interfaces, 2016, 8(42), 28338–28348.

-> Done+ Renumbered references

4) Please use “therefore” instead of “therefor”.

-> Done

5) Please remove the sentence part “as will be shown” in Page 3 Line 138

-> Done

6) Please decide for one style to write the units and apply it for whole manuscript: mV/s or

mV s-1

-> Done

7) Page 4 Line 154: “Cyclic voltammetry offers an insight about the overall characteristics of a

sensor.” It should be “sensor material”.

-> Done

8) Page 9 Part 3.2.1, 3.2.2, and 3.2.3: Results of optimization has to be presented in

“Supporting information”.

-> Thank you for your suggestion. In our opinion the information from the paragraph 3.2.3 is important to underline the difference between sensor and biosensor, highlighting the biosensor performance.

The second reviewer asked more details and explanations for this section.

9) As mentioned before, all Graphs should to be changed massively:

a. Please provide always a y-axis for graphs (figure 2, 3a, etc.)

b. Please use always current density instead of current

c. Again, units should be in the same format: E/V, E(V) or E in V

d. For figure 6, 8: a color legend would be very helpful to understand it easily. e. For figure 7: the number of repetition should be provided for both; figure

caption and manuscript.

-> Done

10) Table 3: according to the caption, all experiments are repeated three-times. However, only a

single value is given. Is this a mean value? If yes, please tell also the deviation…

-> Done

11) Table 4: Please also provide the mean value of three measurements with deviation.

-> Done
